# AUTALASSO: an automatic adaptive LASSO for genome-wide prediction

**DOI:** 10.1186/s12859-019-2743-3

**Published:** 2019-04-02

**Authors:** Patrik Waldmann, Maja Ferenčaković, Gábor Mészáros, Negar Khayatzadeh, Ino Curik, Johann Sölkner

**Affiliations:** 1 Department of Animal Breeding and Genetics, Swedish University of Agricultural Sciences, Box 7023, Uppsala, 750 07 Sweden; 2 Department of Animal Science, Faculty of Agriculture, University of Zagreb, Svetosimunska 25, Zagreb, 10000 Croatia; 3 Division of Livestock Sciences,Department of Sustainable Agricultural Systems,University of Natural Resources and Life Sciences Vienna, Gregor Mendel Str. 33, Vienna, A-1180 Austria

**Keywords:** Genomic selection, GWAS, Regularization, Mathematical optimization, Proximal algorithms

## Abstract

**Background:**

Genome-wide prediction has become the method of choice in animal and plant breeding. Prediction of breeding values and phenotypes are routinely performed using large genomic data sets with number of markers on the order of several thousands to millions. The number of evaluated individuals is usually smaller which results in problems where model sparsity is of major concern. The LASSO technique has proven to be very well-suited for sparse problems often providing excellent prediction accuracy. Several computationally efficient LASSO algorithms have been developed, but optimization of hyper-parameters can be demanding.

**Results:**

We have developed a novel automatic adaptive LASSO (AUTALASSO) based on the alternating direction method of multipliers (ADMM) optimization algorithm. The two major hyper-parameters of ADMM are the learning rate and the regularization factor. The learning rate is automatically tuned with line search and the regularization factor optimized using Golden section search. Results show that AUTALASSO provides superior prediction accuracy when evaluated on simulated and real bull data compared to the adaptive LASSO, LASSO and ridge regression implemented in the popular glmnet software.

**Conclusions:**

The AUTALASSO provides a very flexible and computationally efficient approach to GWP, especially when it is important to obtain high prediction accuracy and genetic gain. The AUTALASSO also has the capability to perform GWAS of both additive and dominance effects with smaller prediction error than the ordinary LASSO.

## Background

Genome-wide prediction (GWP) refers to the idea that regression coefficients, obtained by regressing genomic markers on phenotypic measurements in some training data, can be used to predict phenotypic values without the need to measure the phenotypes in some test data [1–3]. Genome-wide data often consist of several thousands, sometimes millions of markers. Since the number of individuals is usually smaller, in the range of some hundreds to a few thousands, the result is a multivariate high- dimensional statistical issue that is often referred to as the *p*>>*n* problem [4, 5]. The outer product of the design matrix in the ordinary least squares (OLS) estimator is not invertible in these situations and will result in a matrix of rank one. As a consequence, different regularization techniques have been proposed to facilitate in obtaining well-conditioned and unique solutions [6].

Regularization is a mathematical technique to impose prior information on the structure of the solution to an optimization problem. It closely resembles the task of using priors in Bayesian statistics. By formulating regression as a minimization of loss plus penalty function problem it is easy to enforce certain restrictions on the regression coefficients. Ridge regression [7] and the LASSO [8] are two popular regularization methods that only differ in the norms of their penalty functions, i.e. the *ℓ*_2_ and *ℓ*_1_ norms, respectively. It is well established that the LASSO usually results in better prediction accuracy than ridge regression if the predictors display low to moderate correlation between each other [4, 9, 10].

The *ℓ*_1_ norm of the LASSO is special because it results in a convex minimization problem with sparse solutions (many regression coefficients are set to zero) which facilitate computations in large scale situations. A huge number of studies have been devoted to extensions of the lasso in various directions [11]. Generalized Linear Model (GLM), survival and support vector machine versions can be obtained by alternation of the loss function. Modifications of the penalty function have resulted in the elastic net, group and fused LASSO that can handle correlated, structured and time dependent predictor variables. Even more elaborate extensions include sparse additive models and the graphical LASSO [12].

Several optimization algorithms have been developed for the LASSO. The least-angle regression (LAR) algorithm borrows from forward-stepwise regression by updating an active set of regressors via a regularization path [13]. LAR provides relatively fast fitting for sparse data but requires one OLS solution per iteration and is not suitable for all loss functions. Another algorithm that has turned out to be effective is cyclic coordinate descent (CCD). CCD is also an iterative algorithm that updates the regression coefficients by choosing a single coordinate to update, and then perform a univariate minimization over this coordinate. The idea is to compute the OLS coefficients on the partial residuals and apply soft-thresholding to take care of the LASSO contribution to the penalty. CCD efficiently exploits sparsity and can handle large data sets [14].

The rise of big data problems has resulted in a resurgence of first order gradient descent based methods in mathematical optimization [15]. One problem with the LASSO is that the penalty function is nondifferentiable at zero. However, it turns out that the rediscovery of proximal operator techniques has solved this issue. The idea behind the proximal gradient is to decompose the objective function *f* into the sum of one convex, differentiable function *g* and one convex, nondifferentiable function *h*, and then use a proximal map of *h* along with the gradient of *g* to update in each iteration [16]. Proximal versions of the LASSO include the fast iterative soft-thresholding algorithm (FISTA) [17] and the alternating diretion method of multipliers (ADMM) [18]. These methods are fast and can handle large data, but the tuning of the learning rate and the regularization parameter can be tedious.

The purpose of this study is to introduce proximal algorithms, with a special focus on ADMM, into a GWP framework, and then to develop a general approach that automatically finds the optimal values of the learning rate and the regularization parameters of an adaptive LASSO. The learning rate determines how large steps the iterative algorithm takes towards the minimum. Too large steps leads to fewer iterations, but with a high risk to miss the minimum. Too small steps may lead to very many iterations to convergence and therefore also more computing time. The learning rate is optimized using line search with Armijo rule. The level of the regularization parameter in the LASSO regulates how many regression coefficients that are set to zero and is optimized with golden section search. Finally, the method is evaluated on simulated and real GWP data from cattle.

## Results

### Simulated data

**Fig. 1:**
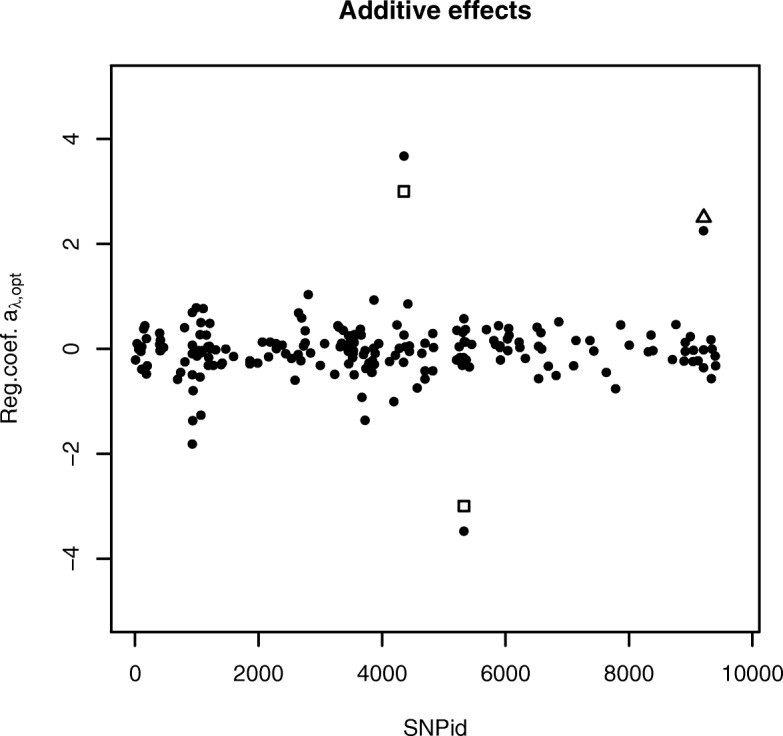
Selected additive genetic effects of the SNPs for the simulated QTLMAS2010 data produced with the AUTALASSO. The two largest estimated effects corresponds well with the simulated effects of the two major controlled QTLs (□) and the third largest effect is close to the additive part of the dominance QTL (△). The number of non-zero variables is 196

**Fig. 2:**
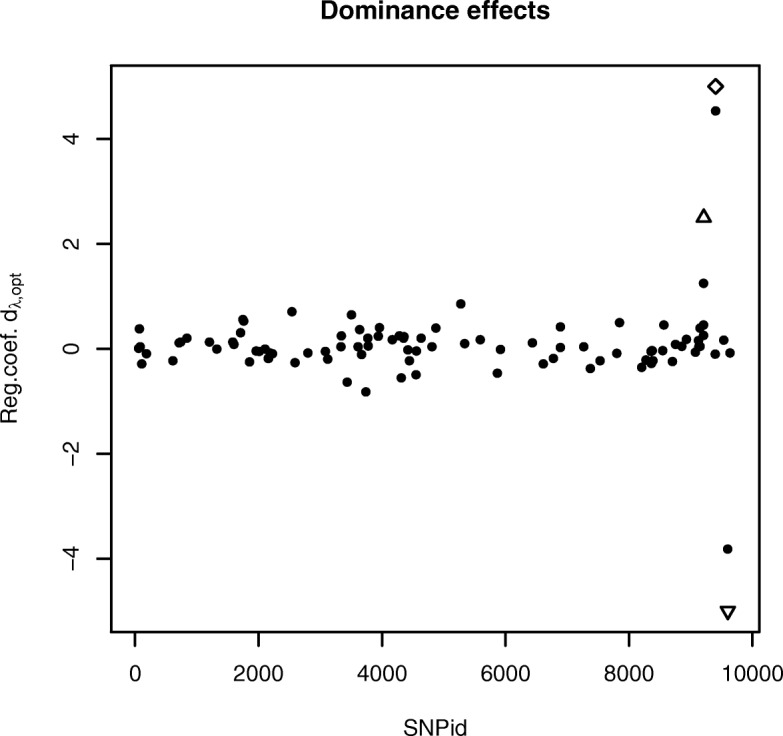
Selected dominance genetic effects of the SNPs for the simulated QTLMAS2010 data produced with the AUTALASSO. The three largest effects corresponds well with the simulated effects of the dominance (△), over-dominance (♢) and under-dominance $(\triangledown)$ QTLs. The number of non-zero variables is 97

For the QTLMAS2010 data we set *ε*_ADMM_=10^−4^ and *ε*_GSS_=10^−2^. Moreover, the initial bracketing points in the GSS procedure were set to *λ*_*a*_=0.0001*λ*_*max*_ and *λ*_*b*_=*λ*_*max*_. In this data set the last generation is used as test data and therefore only one evaluation is needed. The AUTALASSO completed in 190 s and resulted in a MSE_*test*_ of 64.34 and *r*_*test*_ of 0.676. The number of *λ*-values tested to convergence in the GSS algorithm was 21. The additive effects were calculated as $a=-\theta _{\lambda _{opt,gen=0}}+\theta _{\lambda _{opt,gen=2}}$ and the non-zero SNPs are plotted in Fig. 1. It can be seen that the major coefficients corresponds well with the anticipated QTLs, i.e. the two major additive loci and the additive part of the dominance locus. The heterozygote effects equals the dominance effects *d* and selected variables are plotted in Fig. 2. The three most important variables are the dominance, over-dominance and under-domiance loci, respectively. The numbers of selected variables are 196 and 97 for the additive and dominance effects, respectively, in Figs. 1 and 2.

The computing times of the glmnet ALASSO were strongly influenced by the number of *λ*-values, with 7, 56 and 546 s for 100, 1000 and 10,000 values, respectively. However, the influence of varying the number of *λ* on the MSE_*test*_ was small, 64.52, 64.53 and 64.48, respectively. Hence, we decided to use 100 *λ*-values for all analyses. The glmnet LASSO and RR resulted in MSE_*test*_ of 65.73 and 83.07, respectively. The corresponding *r*_*test*_ were for the ALASSO, LASSO and RR 0.675, 0.679 and 0.551, respectively. Hence, the AUTALASSO provides lower MSE_*test*_ regardless of method in glmnet. That the LASSO provides slightly higher *r*_*test*_ than both the AUTALASSO and ALASSO should be interpreted with care (see Discussion).

### Real data

We used the same *ε*-values as for the QTLMAS2010 data in the analyses of the bull data. Moreover, we first ran one analysis of one fold with *λ*_*a*_=0.0001*λ*_*max*_ and *λ*_*b*_=*λ*_*max*_ to get estimates that could guide to a more narrow *λ* interval. The consecutive folds were analysed with *λ*_*a*_=0.0001*λ*_*max*_ and *λ*_*b*_=0.002*λ*_*max*_. The mean timing over the folds was 3020 s and the mean MSE_*test*_ was 11.801 for AUTALASSO. The *r*_*test*_ was estimated to 0.615 for the AUTALASSO. With 100 *λ*-values the mean timing of the glmnet ALASSO was 170 s and mean MSE_*test*_ equal to 13.17. The MSE_*test*_ for the LASSO and RR was 12.65 and 13.20, respectively. The corresponding estimates for *r*_*test*_ were for the ALASSO, LASSO and RR 0.554, 0.579 and 0.554. Hence, the AUTALASSO produces the lowest prediction error and highest prediction accuracy, but is somewhat slower than glmnet. Though it should be noted that glmnet is based on the CCD algorithm and implemented in compiled Fortran which should be faster than Julia.

**Fig. 3:**
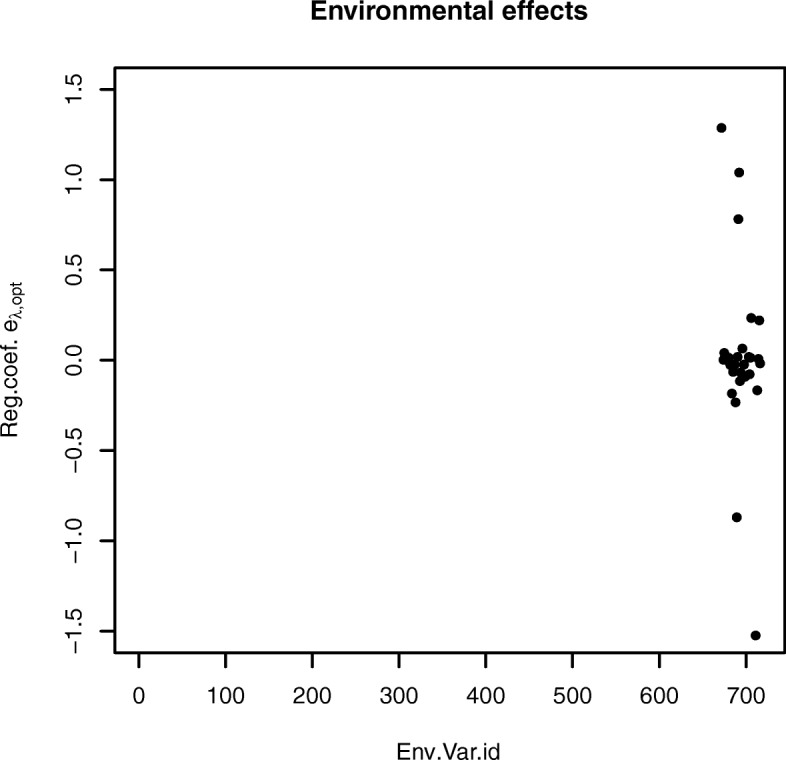
Selected environmental effects for the real Fleckvieh bull data produced with the AUTALASSO. The three largest positive effects on the phenotype are due to age, year 2007 and year 2006, whereas the two most negative are semen collector number 15 and year 2004. The number of non-zero variables is 28

Of the original 716 environmental variables were 28 selected by the AUTALASSO as shown in Fig. 3. None of the 617 repeated measurement indicators were selected. The three highest positive effects were obtained for variables 672 (age), 692 (year 2007) and 691 (year 2006), respectively. Two negative effects also deviate from the others, variable 711 (semen coll. numb.15) and 689 (year 2004).

**Fig. 4:**
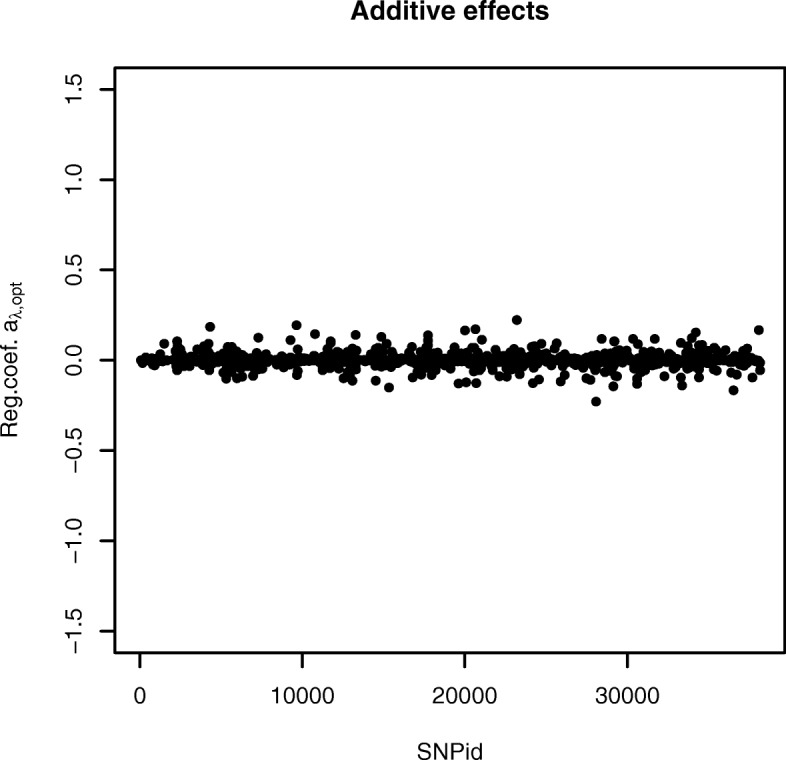
Selected additive genetic SNP effects for the real Fleckvieh bull data produced with the AUTALASSO. The largest positive and negative effects are for SNP 23194 (0.223) and 28087(-0.230), respectively. The number of non-zero variables is 1116

**Fig. 5:**
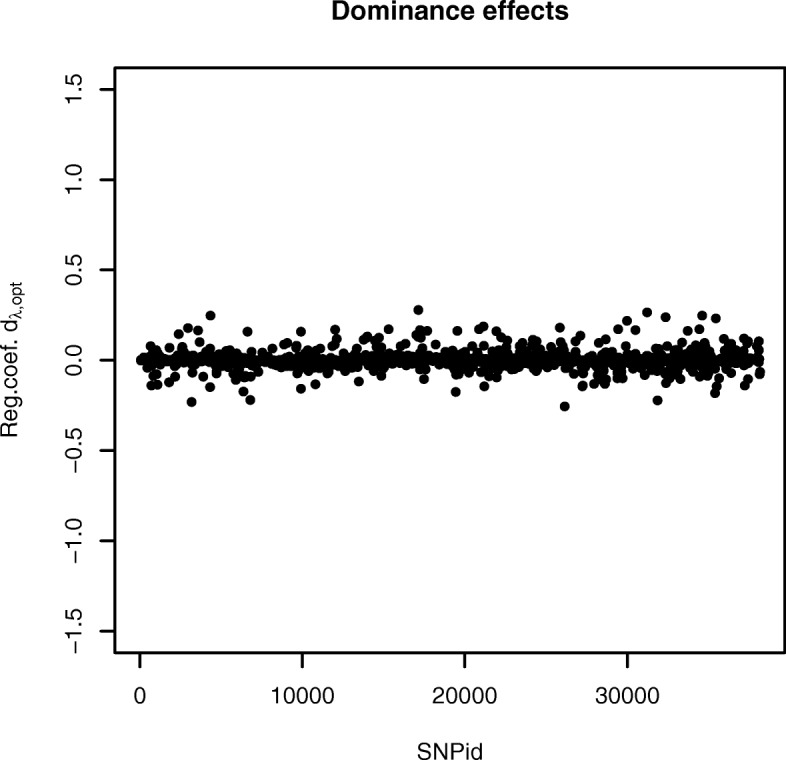
Selected dominance genetic SNP effects for the real Fleckvieh bull data produced with the AUTALASSO. The largest positive and negative effects are for SNP 17125(0.279) and 26154(-0.254), respectively. The number of non-zero variables is 1468

The plots of the selected regression coefficients $\theta _{\lambda _{opt}}$ for the two SNP homozygotes are provided from the authors. The additive and dominance effects were calculated in the same way as for the simulated data. Figure 4 provides a plot of the additive effects of the Fleckvieh bull data. The number of selected additive effects was 1116. The maximum additive effect was 0.223 for SNP 23194, whereas the minimum of -0.230 was found for SNP 28087. Figure 5 provides a plot of the dominance effects of the bull data. The number of selected dominance effects was 1468. The maximum dominance effect was 0.279 for SNP 17125, whereas the minimum of -0.254 was found for SNP 26154.

## Discussion

One of the major obstacles in using high density genomic marker data for prediction purposes is that the number of individuals that can be scored usually is considerably smaller than the number of markers. This situation introduces several problems. For example, when *p*>*n* the ordinary least squares estimates are not unique and will considerably over-fit the data resulting in a low prediction accuracy [4]. Other problems with big genome-wide data sets include spurious random correlations, incidental endogeneity, noise accumulation, and measurement error [19]. Regularization provides an efficient technique to constrain parameters which will lead to unique solutions and often better prediction accuracy. The most successful regularization method is the LASSO because it provides a convex optimization problem with sparse solutions [12]. Here, we have proposed an automatic adaptive LASSO that uses the ADMM algorithm for computing solutions to the objective function, line search for tuning of the learning rate and golden section search for optimization of the regularization parameter.

A large number of methods have been proposed for genome-wide prediction and several papers have evaluated their predictive properties [20, 21]. Regularization approaches include ridge regression, the LASSO and mixture modeling. Their Bayesian counterparts are often referred to as the “Bayesian alphabet” in the genetics literature [22]. The least angle regression version of the LASSO was exploited for GWP in [23] and found to yield better prediction accuracy than genomic BLUP and BayesA. In another study based on glmnet [14], the LASSO, the elastic net, adaptive LASSO and the adaptive elastic net all had similar accuracies but outperformed ridge regression and ridge regression BLUP [10]. In [9], an overview of both the frequentist and Bayesian LASSO is provided. They found the LASSO and adaptive LASSO to be competitive compared to several other methods in evaluations on both simulated and real data. In conclusion, the LASSO seems to be a computationally efficient method that yields good prediction accuracy on GWP data under many situations, but it would be valuable to compile all evaluations into a meta-analysis.

We have calculated both MSE_*test*_ and *r*_*test*_ as measures of model fit and found that they give somewhat contradictory results, especially for the glmnet implementations where the ordinary LASSO had both higher *r*_*test*_ and MSE_*test*_ than the ALASSO. The standard Pearson correlation coefficient is $r_{test} = {\sigma _{\hat {y}_{test}y_{test}}}/\left ({\sigma _{\hat {y}_{test}}\sigma _{y_{test}}}\right)$. When we decompose *r*_*test*_ for the QTLMAS2010 data into its parts, we see that $\sigma _{y_{test}}$ is the same at 10.83 for both methods. However, $\sigma _{\hat {y}_{test}}$ is increased from 5.93 in the LASSO to 6.82 in the ALASSO, and $\sigma _{\hat {y}_{test}y_{test}}$ is increased from 43.556 in the LASSO to 49.86 in the ALASSO. The introduction of the weight factor in the ALASSO increases model complexity which results in decreased model bias, on the expense of an increased variance. However, most importantly, the MSE_*test*_ is reduced. This is an example of the Bias-Variance trade-off that is fundamental in statistical learning [4]. Unfortunately, this phenomenon can complicate the interpretation of *r*_*test*_ as a measure of model fit and it should be used with care.

ADMM is a form of a proximal operator algorithm that has become very popular in mathematical optimization [15]. As described in the “Methods and data” section, we can see that the ADMM is closely related to the augmented Lagrangian method, but does not require a joint minimization which facilitate computations in special applications, for example the LASSO. The ADMM LASSO was first outlined in [18] and since then there have been extensions to for example the clustering LASSO [24] and the generalized LASSO [25]. We have used the least squares (Euclidean) loss function since we perform regression in this study, but it is straightforward to change to a classification loss function (for example logit or hinge loss) in the case of a binary trait.

## Conclusions

The AUTALASSO provides a very flexible and computationally efficient approach to GWP, especially in situations were it is important to obtain high prediction accuracy and genetic gain. The algorithm automatically tunes the learning rate with line search and the regularization factor using Golden section search. The results show that the prediction error is smaller for the AUTALASSO when compared with the adaptive LASSO, the ordinary LASSO and ridge regression implemented in the popular glmnet package, albeit at the expense of some increased computing time. It is also shown that important SNP markers can be identified, both with underlying additive and dominance effects. The implementation in Julia is easy to modify, and we have hope that future versions of the AUTALASSO will fully capitalize on the distributed computing facilities of the ADMM algorithm.

## Methods and data

### ADMM adaptive LASSO

The LASSO was introduced by [8] and the objective function of the optimization problem consists of an ordinary squared *ℓ*_2_ (Euclidean) norm loss function and an *ℓ*_1_ norm penalty function

1 $$ {\text{minimize }}f(\beta)+g(\beta)  $$

where $f(\beta)=(1/2)\left \| X\beta -y \right \|_{2}^{2}$ and *g*(*β*)=*λ*∥*β*∥_1_. In ADMM form (see Appendix), the LASSO becomes

2 $$ \begin{aligned} &{\text{minimize }}f(\beta)+g(\theta)\\ &{\text{subject to }}\beta-\theta=0. \end{aligned}  $$

The ADMM algorithm then is the following iterative procedure

3 $$ \begin{aligned} &\beta^{t+1}=\left(X^{T}X+{\rho}I\right)^{-1}+\left(X^{T}y+{\rho}\left(\theta^{t}-u^{t}\right)\right)\\[-2pt] &\theta^{t+1}=S_{{\lambda\rho}}\left(\beta^{t+1}+u^{t}\right)\\[-2pt] &u^{t+1}=u^{t}+\beta^{t+1}-\theta^{t+1} \end{aligned}  $$

where *ρ*>0 is the learning rate, *λ*>0 is the regularization parameter and *S*_*λρ*_(*ν*)=(*ν*−*λρ*)_+_−(−*ν*−*λρ*)_+_ is the soft-thresholding operator that depends on both *ρ* and *λ*. It is now possible to rewrite this algorithm using proximal operators (see Appendix)

4 $$ \begin{aligned} &\beta^{t+1}=\operatorname{prox}_{\rho f}\left(\theta^{t}-u^{t}\right)\\[-2pt] &\theta^{t+1}=\operatorname{prox}_{{\lambda}{\rho}g}\left(\beta^{t+1}+u^{t}\right)\\[-2pt] &u^{t+1}=u^{t}+\beta^{t+1}-\theta^{t+1}, \end{aligned}  $$

which is iterated to convergence determined by

5 $$ \begin{aligned} &\left\| \beta^{t+1}- \theta^{t+1}\right\|_{\infty}\leq \epsilon_{\text{ADMM}}\left(1+\left\| \mu^{t+1}\right\|_{\infty}\right) \end{aligned}  $$

for tolerance parameter *ε*_ADMM_. The choice of *ε*_ADMM_ can have a dramatic influence on the number of iterations to convergence and we recommend to set it between 10^−3^ and 10^−4^.

In [26], it was shown that the LASSO can be inconsistent for variable selection under some situations and the adaptive LASSO was suggested in order to reduce bias and full-fill oracle properties (i.e that the estimator satisfies support and $\sqrt {n}$-estimation consistency). The idea behind the adaptive LASSO is to use variable specific weights $\hat {w}=1/|{\hat {\beta }}|^{\gamma }$, where ${\hat {\beta }}$ is estimated based on some pilot run and *γ*>0 a tuning parameter. The OLS estimates of ${\hat {\beta }}$ are not defined when *p*>*n*, but univariate marginal regression (or covariance) coefficients calculated as ${\hat {\beta } = X^{T}y/n}$ can be used and yield good recovery properties under most situations [27]. Moreover, the adaptive lasso penalty can be seen as an approximation to the *ℓ*_*q*_ penalties with *q*=1−*γ*. Hence, *γ*=1 approximates the *ℓ*_0_ penalty [12]. This formulation is a great advantage since the penalty function is still convex and the only part of the objective function that needs to be changed in the adaptive LASSO is $g^{*}(\beta)={\lambda }\left \|\hat {w}\beta \right \|_{1}$.

### Automatic tuning of the learning rate and the regularization factor

Many procedures have been proposed for tuning of the learning rate *ρ* in gradient decent algorithms. Since the proximal gradient is a special case of projected gradients methods we can use the fact that line search with Armijo rule can be used for optimization of the learning rate in this form of constrained optimization [15]. Given that the projected gradient starts from the current iterate of *β*^*t*^, moves in the direction of the negative gradient of the loss function *β*^*t*^−*ρ*∇*f*(*β*^*t*^) and then performs a projection back to the convex constraint set that holds *θ*^*t*^, a line search can be formulated by iteratively decreasing *ρ*^*k*^ until

6 $$ \begin{aligned} &f\left(\theta^{t}\right) > f\left(\beta^{t}\right)-{\nabla}f\left(\beta^{t}\right)^{T}\beta+\left(1/2{\rho}^{k}\right)\left\| \beta^{t}\right\|_{2}^{2} \\ \end{aligned}  $$

no longer holds. The gradient is calculated as ∇*f*(*β*^*t*^)=*X*^*T*^(*Xβ*^*t*^−*y*). A suitable choice can be to start with *ρ*^0^=1 and decrease *ρ*^*k*^ by a factor of 0.5 each *k*th iteration until *ρ*^*K*^ is found. A new *ρ*^*K*^ is then used in each *t*th ADMM update.

The bias-variance tradeoff in statistical learning makes it necessary to use test (or validation) data to avoid under- and overfitting [4]. A common approach to find the minimum test MSE of a LASSO evaluation is to calculate the value of the regularization factor where all regression coefficients of the training data are zero, i.e. *λ*_*max*_=∥*X*^*T*^*y*∥_*∞*_, and then to evaluate the MSE_test_ along a path of equally spaced and decreasing *λ*-values until an arbitrarily choosen *λ*_*min*_ is reached [14]. Often, this amounts to fitting the LASSO for at least 100 *λ*-values, and the precision in finding the optimum depends on how well the path covers the shape of the test error function around the minimum.

Another option is to optimize the *λ*-value using a formal optimization algorithm that minimize the convex test error function. Here we propose to use the Golden section search (GSS) which is a technique for finding the minimum of a function by successively narrowing the range of values inside which the minimum is known to exist [28]. The GSS algorithm is described as follows:

1. Set two bracketing points, for example *λ*_*a*_=0.001*λ*_*max*_ and *λ*_*b*_=*λ*_*max*_.
2. Fit the ADMM LASSO with line search to the training data for *λ*_*a*_ and *λ*_*b*_ to obtain $\theta _{\lambda _{a}}$ and $\theta _{\lambda _{b}}$ which are used to predict the squared test errors
  7 $$ \begin{aligned} &\text{SE}_{test,\lambda_{a}}=1/2\left\|X_{test}\theta_{\lambda_{a}}-y_{test}\right\|_{2}^{2}\\ &\text{SE}_{test,\lambda_{b}}=1/2\left\|X_{test}\theta_{\lambda_{b}}-y_{test}\right\|_{2}^{2}.\\ \end{aligned}  $$
3. Set the golden section ratio to $\varphi = \left (1+\sqrt {5}\right)/2$.
4. Let *λ*_*c*_=*λ*_*b*_−(*λ*_*b*_−*λ*_*a*_)/*φ* and *λ*_*d*_=*λ*_*a*_+(*λ*_*b*_−*λ*_*a*_)/*φ*.
5. Compute both $\text {SE}_{test,\lambda _{c}}$ and $\text {SE}_{test,\lambda _{d}}$ following (7) in the first iteration. For iterations >1 calculate $\text {SE}_{test,\lambda _{c}}$ if *flag*=1 and $\text {SE}_{test,\lambda _{d}}$ if *flag*=0.
6. If $\text {SE}_{test,\lambda _{c}}<\text {SE}_{test,\lambda _{d}}$ then move *λ*_*d*_→*λ*_*b*_, $\text {SE}_{test,\lambda _{c}} \rightarrow \text {SE}_{test,\lambda _{d}}$, $\theta _{\lambda _{c}} \rightarrow \theta _{\lambda _{d}}$. Set *flag*=1. Compute new *λ*_*c*_=*λ*_*b*_−(*λ*_*a*_−*λ*_*b*_)/*φ* and *λ*_*d*_=*λ*_*a*_+(*λ*_*b*_−*λ*_*a*_)/*φ*.
7. Otherwise move *λ*_*c*_→*λ*_*a*_, $\text {SE}_{test,\lambda _{d}} \rightarrow \text {SE}_{test,\lambda _{c}}$, $\theta _{\lambda _{d}} \rightarrow \theta _{\lambda _{c}}$. Set *flag*=0.. Compute new *λ*_*c*_=*λ*_*b*_−(*λ*_*a*_−*λ*_*b*_)/*φ* and *λ*_*d*_=*λ*_*a*_+(*λ*_*b*_−*λ*_*a*_)/*φ*.
8. Repeat 5-7 until (|*λ*_*d*_−*λ*_*c*_|/((*λ*_*c*_+*λ*_*d*_)/2))<*ε*_GSS_.
9. Calculate *λ*_*opt*_=(*λ*_*c*_+*λ*_*d*_)/2 and perform a final ADMM run to get $\theta _{\lambda _{opt}}$ and $\text {SE}_{test,\lambda _{opt}}$.

The value to choose for the convergence tolerance *ε*_GSS_ can have a large effect on computing time. For a relatively good balance between precision and computing time, *ε*_GSS_ can be set between 0.01 and 0.001. The start values of *θ* and *β* also influence computing time in each ADMM run. Hence, the *θ* and *β* fits in step 5 of the GSS algorithm are re-used in next iteration to obtain warm-starts, but the *u*-vector is set to 0. Warm-starts have been shown to reduce the number of iterations to convergence in ADMM considerably [29].

### Simulated data

The original data was produced for the QTLMAS2010 work-shop [30]. The total number of individuals is 3226 and they are structured in a pedigree with 5 generations. The pedigree is founded by 20 individuals (5 males and 15 females), and created assuming that each female mates once and gives birth to approximately 30 progeny. Five autosomal chromosomes of length 100Mbp were simulated. A neutral coalescent model was used to simulate the SNP data. The procedure created 10,031 markers, including 263 monomorphic and 9768 biallelic SNPs.

The continuous quantitative trait was created from 37 QTLs, including 9 controlled genes and 28 random genes. The controlled QTLs included two pairs of epistatic genes with no individual effects, 3 maternally imprinted genes and two additive major genes with effects of -3 and 3. The random genes were selected from the simulated SNPs and then their effects were sampled from a truncated normal distribution, and accepted if the absolute value of the additive effect was smaller than 2. The resulting additive effects of the random genes varied between -1.98 and 1.93. Each simulated QTL was surrounded by 19-47 polymorphic SNPs (MAF >0.05) positioned within 1 Mb distance from the QTL. 364 SNPs were in moderate to high LD with the QTLs.

In addition to the original data, one dominance locus was positioned at SNP number 9212 on chromosome 5 by giving the heterozygote (1) an effect of 5.00 and the upper homozygote (2) a value of 5.01. One over-dominance locus was produced at SNP 9404 by assigning the heterozygote an effect of 5.00, and lower homozygote (0) the effect of -0.01 and upper homozygote (2) the effect of 0.01. Finally, one under-dominance loci was created at SNP id 9602 by assigning a value of -5.00 to the heterozygote, and lower homozygote (0) the effect of -0.01 and upper homozygote (2) the effect of 0.01. The values of the genotypes of these new QTLs were added to the original *y*-values. MAF cleaning was performed at the 0.01 level so the final sample of SNPs with 0,1,2 coding was 9723. The SNPs were converted into one-hot encoding, i.e. indicator variables for each genotype. Hence, the final number of genomic markers was 29169. Data from individual 1 to 2326 were used as training data and from individual 2327 to 3226 (the 5th generation) as test data.

### Real data

The SNP genotype data comes from the bovine 50k Beadchip and the phenotypes measured were total number of spermatozoa in ejaculates during routine artificial insemination programs, both obtained from Austrian Fleckvieh bulls [31]. Sperm quality data were obtained from 3 Austrian AI stations: Gleisdorf station in Styria (7704 ejaculates, 301 bulls sampled from 2000 to 2010), Hohenzell station in Upper Austria (16671 ejaculates, 309 bulls sampled from 2000 to 2009), and Wieselburg station in Lower Austria (15514 ejaculates, 293 bulls sampled from 2000 to 2009). In addition to year and station were also age of bull (13 - 161 months), month of year (January - December), period between 2 successive ejaculates (0 - 60 days) and collector (1 - 17) used as input variables. All variables, except age of bull and period between successive ejaculates, were transformed into 0,1 indicator variables (one-hot encoding). Age of bull and period between successive ejaculates were standardized to mean 0 and SD 1, whereas the phenotype total number of spermatozoa was mean centered. The final number of environmental variables was 716, where the first 617 corresponds to bull repeated measurements indicators.

1799 Austrian Fleckvieh bulls were genotyped using the bovine SNP50 Beadchip v1 (Illumina) which contains 54001 SNPs (50k). Only autosomal SNPs that were assigned to a chromosome were used. Missing SNP data were imputed using FImpute [32] and converted to 0,1 and 2 code. SNPs with MAF smaller than 0.01 were removed resulting in a total of 38871 SNPs. These SNPs were also converted into indicator variables which produced 116613 markers. Bulls without phenotype measures were also removed. The final number of bulls was 671 with a total of 21567 phenotype measures. The data was randomly divided into 10 repeats (corresponding to 10-fold cross-validation) of training data with 15097 observations and test data with 6470 observations.

### Implementation

The full automatic adaptive LASSO (AUTALASSO) was implemented in Julia version 0.6 [33] and uses the ProximalOperators package for the proximal steps in the ADMM algorithm. The Julia code used for the QTLMAS2010 data is provided at: https://github.com/patwa67/AUTALASSO.

The prediction accuracy of the AUTALASSO was compared with the R implementation of an adaptive LASSO (ALASSO), the ordinary LASSO (LASSO) and ridge regression (RR) using the glmnet package with default settings [14]. For comparative purposes were both MSE_*test*_ and Pearson correlation coefficient (*r*_*test*_) calculated. The evaluations were performed on an Intel Xeon E5 4-Core 3.7GHz with 256GB RAM.

## Appendix

### Proximal operators

A proximal operator prox*f* is used to evaluate a closed and proper convex function *f*(*β*) of a specific optimization subproblem that is assumed to be easier to solve than the original problem. By iteratively evaluating proximal operators on subproblems, a proximal algorithm converges to the solution of the original problem [34]. The proximal operator is defined as

8 $$ \operatorname{prox}_{f}(\theta) = \underset{\beta}{\operatorname{argmin}} \left(f(\beta)+(1/2)\left\| \beta -\theta \right\|_{2}^{2}\right)   $$

where ∥·∥_2_ is the Euclidean norm, and *β* and *θ* are vectors of length *p*. The right hand side of the argument is strongly convex so it has a unique minimizer for every *θ*∈*R*^*p*^. A scaled version of (8) is obtained by introducing parameter *λ*>0 resulting in *λf* where (1/2) is replaced by (1/2*λ*). This definition indicates that prox*f*(*θ*) is a point that compromises between minimizing *f* and being close to *θ*. *λ* can be seen as a trade-off parameter between these two terms. Another key property is for separable sum functions *f*(*β*,*θ*)=*g*(*β*)+*h*(*θ*) where splitting leads to

9 $$ \operatorname{prox}_{f}(\beta,\theta) = \operatorname{prox}_{g}(\beta)+\operatorname{prox}_{h}(\theta).   $$

Finally we note that there is a near relationship between proximal operators and gradient methods where

10 $$ \operatorname{prox}_{\lambda f}(\beta) \approx \beta-\lambda \nabla f(\beta)   $$

when *λ* is small and *f*(*β*) is differentiable. In this formulation, ∇ denotes the gradient and *λ* is an equivalent to the learning rate of a gradient optimizer [16].

### Alternating direction method of multipliers (ADMM)

The alternating direction method of multipliers (ADMM) is a simple but powerful algorithm that is appropriate for distributed convex optimization, and specifically to problems in statistics and machine learning. It is based on decomposition of a large global problem into small local coordinated subproblems to find a solution [18].

In order to understand the ADMM, it is useful to introduce the methods of dual ascent and augmented Lagrangians. For the convex optimization problem

11 $$ \begin{aligned} &\underset{\beta}{\text{minimize }}f(\beta)\\ &{\text{subject to }}X\beta = y \end{aligned}  $$

the Langrangian will be

12 $$ L(\beta,\theta)=f(\beta)+\theta^{T}(X\beta - y)  $$

and the dual function is

13 $$ g(\theta)=\underset{\beta}{\text{inf }}L(\beta,\theta)=-f^{*}\left(-X^{T}\theta\right)-y^{T}\theta  $$

where *X* is a matrix of size *n*×*p*, *y* is a vector of length *n*, *θ* is the dual variable and *f*^∗^ is the convex conjugate of *f*. The dual problem is to maximize *g*(*θ*). In dual ascent the dual problem is solved using gradient ascent. If the dual function is differentiable the following iterating scheme describes dual ascent

14 $$ \begin{aligned} &\beta^{t+1}=\underset{\beta}{\operatorname{argmin}}L\left(\beta,\theta^{t}\right)\\ &\theta^{t+1}=\theta^{t}+\rho\left(X\beta^{t+1}-y\right) \end{aligned}  $$

where *ρ*>0 is the learning rate and *t* is the iteration number. The first step of (14) is a *β*-minimization step and the second step is a dual variable update. If *ρ*>0 is adequately chosen and several assumptions hold, then *β*^*t*^ converges to an optimal point and *θ*^*t*^ converges to an optimal dual point.

Augmented Lagrangian methods were partly developed to make the dual ascent method more robust in situations where the objective function lacks strict convexity. The augmented Lagrangian for (11) is

15 $$ L_{\lambda}(\beta,\theta)=f(\beta)+\theta^{T}(X\beta - y)+(\lambda/2)\left\| X\beta - y \right\|_{2}^{2}  $$

where *λ* is the penalty parameter. The augmented Lagrangian can be used in an iterative procedure denoted the method of multipliers

16 $$ \begin{aligned} &\beta^{t+1}=\underset{\beta}{\operatorname{argmin}}L_{\lambda}\left(\beta,\theta^{t}\right)\\ &\theta^{t+1}=\theta^{t}+\lambda\left(X\beta^{t+1}-y\right) \end{aligned}  $$

which is the same as dual ascent, except for the augmented part of the Lagrangian and the use of *λ* as step size. The method of multipliers has better convergence properties than dual ascent.

By extending the parameter space into *β* and *θ*, the optimization problem for the ADMM algorithm can now be rewritten as

17 $$ \begin{aligned} &\underset{\beta}{\text{minimize }}f(\beta)+g(\theta)\\ &{\text{subject to }}X\beta +Z\theta= y \end{aligned}  $$

where the augented Lagrangian is

18 $$ \begin{aligned} L_{\lambda}(\beta,\theta,\mu)&=f(\beta)+g(\theta)+\mu^{T}(X\beta+Z\theta- y)\\ &+(\lambda/2)\left\| X\beta+Z\theta-y \right\|_{2}^{2} \end{aligned}  $$

and the iterative ADMM algorithm becomes

19 $$ \begin{aligned} &\beta^{t+1}=\underset{\beta}{\operatorname{argmin}}L_{\lambda}\left(\beta,\theta^{t},\mu^{t}\right)\\ &\theta^{t+1}=\underset{\theta}{\operatorname{argmin}}L_{\lambda}\left(\beta^{t+1},\theta,\mu^{t}\right)\\ &\mu^{t+1}=\mu^{t}+\lambda(X\beta^{t+1}+Z\theta^{t+1}-y) \end{aligned}  $$

Noting that the primal residual is *e*=*Xβ*+*Zθ*−*y* and the scaled dual variable is *u*=(1/*λ*)/*μ* a scaled version of (19) can be obtained as

20 $$ {{} \begin{aligned} &\beta^{t+1}=\underset{\beta}{\operatorname{argmin}}\left(f(\beta)+(\lambda/2)\left\| X\beta+Z\theta^{t}-y+u^{t} \right\|_{2}^{2}\right)\\ &\theta^{t+1}=\underset{\theta}{\operatorname{argmin}}\left(g(\theta)+(\lambda/2)\left\| X\beta^{t+1}+Z\theta-y+u^{t} \right\|_{2}^{2}\right)\\ &u^{t+1}=u^{t}+X\beta^{t+1}+Z\theta^{t+1}-y \end{aligned}}  $$

and *r*^*t*+1^=*λX*^*T*^*Z*(*θ*^*t*+1^−*θ*^*t*^) can be interpreted as a dual residual. It can be shown that both *r*^*t*^ and *e*^*t*^ approach zero at convergence [16].
